# Does ChatGPT have a typical or atypical theory of mind?

**DOI:** 10.3389/fpsyg.2024.1488172

**Published:** 2024-10-29

**Authors:** Margherita Attanasio, Monica Mazza, Ilenia Le Donne, Francesco Masedu, Maria Paola Greco, Marco Valenti

**Affiliations:** ^1^Department of Biotechnological and Applied Clinical Sciences, University of L’Aquila, L’Aquila, Italy; ^2^Reference Regional Centre for Autism, Abruzzo Region, Local Health Unit, L’Aquila, Italy

**Keywords:** large language models, ChatGPT, artificial intelligence, autism spectrum disorder, Theory of mind, emotion

## Abstract

In recent years, the capabilities of Large Language Models (LLMs), such as ChatGPT, to imitate human behavioral patterns have been attracting growing interest from experimental psychology. Although ChatGPT can successfully generate accurate theoretical and inferential information in several fields, its ability to exhibit a Theory of Mind (ToM) is a topic of debate and interest in literature. Impairments in ToM are considered responsible for social difficulties in many clinical conditions, such as Autism Spectrum Disorder (ASD). Some studies showed that ChatGPT can successfully pass classical ToM tasks, however, the response style used by LLMs to solve advanced ToM tasks, comparing their abilities with those of typical development (TD) individuals and clinical populations, has not been explored. In this preliminary study, we administered the Advanced ToM Test and the Emotion Attribution Task to ChatGPT 3.5 and ChatGPT-4 and compared their responses with those of an ASD and TD group. Our results showed that the two LLMs had higher accuracy in understanding mental states, although ChatGPT-3.5 failed with more complex mental states. In understanding emotional states, ChatGPT-3.5 performed significantly worse than TDs but did not differ from ASDs, showing difficulty with negative emotions. ChatGPT-4 achieved higher accuracy, but difficulties with recognizing sadness and anger persisted. The style adopted by both LLMs appeared verbose, and repetitive, tending to violate Grice’s maxims. This conversational style seems similar to that adopted by high-functioning ASDs. Clinical implications and potential applications are discussed.

## 1 Introduction

Theory of Mind (ToM), namely the ability to understand and infer one’s own and others’ mental states in terms of beliefs, intentions, thoughts, emotions, and desires (Frith and Frith, 2003; Mazza et al., 2024), represents a crucial skill for an individual’s social life. As one of the most complex and sophisticated abilities of humans, it represents a daunting challenge in the development of modern artificial intelligence (AI). In recent years, Large Language Models (LLMs), such as Generative Pre-trained Transformer (GPT) models, have shown remarkable natural language processing capabilities and a potential ability to simulate human behavioral and cognitive patterns (Sartori and Orrù, 2023). ChatGPT is a chatbot based on LLM and specializes in conversation with human users thanks to machine learning algorithms. Although ChatGPT can successfully generate accurate theoretical and inferential information in various fields, its ability to exhibit adequate ToM, similar to that of typically developing humans, is a topic of debate and interest in experimental clinical psychology (Marchetti et al., 2023; Tavella et al., 2024; Trott et al., 2023). For example, in the study by Strachan et al. (2024), GPT-3.5 scored significantly below human levels in the irony comprehension test and the faux pas test. Kosinski (2023) showed that the more advanced models, such as ChatGPT - 4, unlike the smaller models, can solve false belief tasks by achieving similar performance as 6-year-old children. Other studies have shown mixed results, varying based on the task used, the prompt provided and the questions asked (Brunet-Gouet et al., 2023). A recent study conducted by Barattieri di San Pietro et al. (2023) compared the pragmatic language capabilities of ChatGPT-3.5 with those of humans and showed that ChatGPT’s performance was similar to humans but with a drop in recognition of physical metaphors, understanding of humor, and violation of Grice’s maxims (Grice, 1975), supporting a tendency to the artificiality of response (Marchetti et al., 2023).

Regarding the affective dimension of ToM and empathic abilities, studies have shown that LLMs are potentially able to simulate some aspects of empathy, although their responses often appear repetitive or too general (Chen et al., 2023; Schaaff et al., 2023; Sorin et al., 2023). Elyoseph et al. (2023) demonstrated that ChatGPT can generate responses characterized by appropriate emotional awareness, including successfully identifying and describing emotions.

Difficulties in ToM, emotional awareness skills, and pragmatic language are well-documented in the literature as defining characteristics of clinical populations, most notably Autism Spectrum Disorder (ASD) (Baron-Cohen et al., 2015; Boada et al., 2020; Deliens et al., 2018; Mazza et al., 2022, 2024). ASD is a complex neurodevelopmental disorder presenting deficits in communication and social interaction and patterns of restricted and repetitive behavior and interests (American Psychiatric Association, 2013). A recent study by Mazza et al. (2022) showed that the observation of response style in advanced ToM tasks helps to distinguish between clinical and non-clinical populations and supports the differential diagnosis between ASD and Schizophrenia Spectrum Disorders.

To our knowledge, the response style used by LLMs to solve advanced ToM tasks has not been explored in detail. Furthermore, studies addressing the ToM abilities of LLMs with human participants, including clinical populations, are still lacking in the literature. Based on these assumptions and taking into account that studies exploring the potential applications of AI and LLMs in clinical and mental health settings are growing (Thirunavukarasu et al., 2023a, 2023b), we preliminary investigated and discussed whether the reasoning style used by ChatGPT for mentalizing tasks overlaps with that used by Typical Development (TD) or ASD populations.

## 2 Materials and methods

### 2.1 Participants

#### 2.1.1 Human participants

Two different groups of TD individuals recruited by opportunity from local structures and organizations participated in the study in two separate sessions: (1) 54 healthy individuals (39 females and 15 males; mean chronological age 20.8 ± 2.35;) who completed the Advanced Theory of Mind Task (Blair and Cipolotti, 2000; Mazza et al., 2022; Prior et al., 2003); (2) 54 healthy individuals (38 females and 16 males; mean chronological age 20.6 ± 2.08) who completed the Emotion Attribution Task (Blair and Cipolotti, 2000; Prior et al., 2003). The exclusion criteria considered were the presence of neurological diseases, psychiatric disorders, cognitive disorders, substance disorders, and head trauma.

The ASD group was composed of 51 individuals with Level-1 ASD (11 females and 40 males; mean chronological age 22.4 ± 7.87; IQ mean 99.7 ± 12.9), recruited by the Reference Regional Centre for Autism in L’Aquila, Italy. The diagnosis was formulated by clinical experts according to DSM-5 criteria (American Psychiatric Association, 2013) and using Autism Diagnostic Observation Schedule-Version 2 (Lord et al., 2012). The exclusion criteria considered were the presence of intellectual disability, epilepsy, speech disorders, and psychiatric comorbidities.

#### 2.1.2 Large language models

We used ChatGPT (OpenAI, San Francisco) which is one of the most popular and free LLM online. Our experiments were conducted using the 22 January 2024 version of ChatGPT 3.5 and the 26–27 February 2024 version of ChatGPT- 4.

### 2.2 Measures

- *Advanced Theory of Mind Task (A-ToM)* (Blair and Cipolotti, 2000; Mazza et al., 2022; Prior et al., 2003) is an Italian adaptation of a cognitive ToM task (i.e., Strange Stories; Happé, 1994) that consists of 13 stories describing real events; for a correct interpretation, the task requires the subject to go beyond the literal meaning of the text and draw an inference about the mental state of the story’s protagonist. Each story represents a different type of mental state attribution, namely: fiction, persuasion, joke, lie, white lie, equivocation, irony, double bluff and sarcasm (Mazza et al., 2022). Each story is followed by two questions: a comprehension question (e.g., “Was what X said true?”) and a justification question (e.g., “Why did X say that?”). For each story, a score of 1 is assigned if both the comprehension and justification questions are answered correctly, otherwise a score of 0 is awarded. An answer to the justification question is considered correct if it contains a physical attribution (i.e., answers that refer to non-mental events, such as physical appearance, action of an object, physical events, and results) or mental attribution (i.e., responses that contain correctly identified thoughts, feelings, desires or figures of speech). The total score can vary from 0 to 13, where a higher score corresponds to a better understanding of the mental state of others.
- *Emotion Attribution Task* (EAT) (Blair and Cipolotti, 2000; Prior et al., 2003) is a ToM affective task that assesses the ability to attribute emotional states to others. It consists of 58 stories that describe emotional situations that arouse attributions of positive and negative emotions, in particular: 10 stories arouse happiness, 10 sadness, 10 fear, 12 embarrassment, 3 disgust, 10 anger, and 3 envy. In the task, the participant is asked to provide the emotion that best describes the feeling experienced by the protagonist of the story. The encoding takes place through a list of correct answers (target emotions and synonyms) for each story. The correct answer is coded as 1; otherwise, it is coded as 0. A higher score is equivalent to a better understanding of the relative attribution of emotions.

### 2.3 Procedure

Human participants were evaluated individually using a paper and pencil procedure. The experimental protocol was approved by the local Ethics Committee (NHS Local Health Unit- Azienda Sanitaria Locale 1, protocol nr. 0052505/21). The study was conducted according to the principles established by the Declaration of Helsinki and informed consent was obtained from each participant before the test was administered.

To test the performance of ChatGPT 3.5 and ChatGPT- 4, the same test protocols used for testing human participants were administered. The stories were then placed in the ChatGPT “message box” and asked to answer the questions as planned for the two tests. The questions and answers were in Italian. Specifically, we started two different chats for the two tests and entered each story sequentially. In the EAT both ChatGPT-3.5 and ChatGPT-4 needed an additional prompt (translated from Italian: “*Try to be as precise as possible in indicating the emotion the protagonist will feel”*), to accomplish the task and try to arrive at a more precise answer. This additional specification was used for each story, after receiving an initial response (without additional prompt). Our evaluations were based on the answers given after the additional request was entered. ChatGPT’s responses were evaluated following the same criteria used for humans and were independently coded by 3 team researchers, discussing any disagreements until unanimity was reached.

### 2.4 Statistical analysis

#### 2.4.1 Comparison between TD and ASD groups

The differences between the ASD and TD groups on chronological age, performance on A-ToM and EAT were analyzed through the Mann–Whitney test.

#### 2.4.2 ChatGPT performances

The performance of ChatGPT-3.5 and ChatGPT-4 was analyzed as two single cases, calculating the total raw scores (correct and incorrect responses) and the proportion of accuracy for each test. The performances of the two LLMs were compared using a two-sample proportion z-test.

#### 2.4.3 Comparison between ChatGPT against human performances

Mean scores and mean proportions of accuracy for each task were calculated for each group (TD and ASD). The proportions of correct responses of ChatGPT-3.5 and ChatGPT-4 were compared with those of the ASD and TD groups using a one-sample proportion test (binomial test).

## 3 Results

### 3.1 Comparison between TD and ASD groups

The TD group and the ASD group who completed the A-ToM test did not show differences regarding chronological age (*U* = 1,130, *p* = 0.11). Our results showed a significant difference in the Total Score of A-ToM (*U* = 737, *p* < 0.001), where the ASD group reported lower scores than the TD group.

Regarding the EAT test, our results showed that the ASD group and the TD group did not differ in chronological age (*U* = 1,176, *p* = 0.19). We found a significant difference in Total Score (U = 931, *p* = 0.004) and in Embarrassment Score (*U* = 770, *p* < 0.001), where the ASD group performed worse than the TD group. No other significant differences were found between groups on the EAT test.

The results are reported in Table 1.

**Table 1:** Comparison between TD and ASD groups on the A-ToM and EAT tests.

	TD (*n* = 54)	ASD (*n* = 51)	*U*	*p*
A-ToM	11.52 (1.26)	9.45 (2.92)	737	**<0.001**
EAT				
Sadness	6.31 (2.28)	6.31 (2.07)	1,352	0.87
Fear	8.04 (1.75)	7.57 (2.21)	1,244	0.38
Embarrassment	8.07 (1.84)	5.24 (3.68)	770	**<0.001**
Disgust	2.50 (0.79)	2.31 (0.81)	1,172	0.13
Happiness	8.13 (1.69)	7.37 (2.64)	1,230	0.34
Anger	5.31 (2.29)	4.92 (2.51)	1,265	0.47
Envy	1.78 (1.00)	1.51 (1.22)	1,227	0.32
EAT Total Score	40.15 (7.57)	35.24 (9.25)	931	**0.004**

Significant comparisons are reported in bold.

### 3.2 ChatGPT performances

In the A-ToM, ChatGPT-3.5 answered 11/13 (84.6%) questions correctly. Specifically, it failed one comprehension question (Story 2- Persuasion) and one justification question (Story 11- Double Bluff). On the contrary, ChatGPT-4 answered all questions correctly, both comprehension and justification, obtaining a total score of 13/13 (100%). The results of the two-sample proportion z-test (z − 1.47, *p* = 0.14) showed no significant differences in the performance of the ChatGPT-3.5 and the ChatGPT-4 on A-ToM.

In the EAT ChatGPT-3.5 scored 31/58 (53.4%), with more errors in identifying the emotions of anger, envy, and sadness. We observed an overall better performance in ChatGPT- 4 with a Total Score of 38/58 (65.52%), although not statistically significantly different than ChatGPT-3.5 (z − 1.32, *p =* 0.18). In the single emotions, no statistically significant differences emerged between ChatGPT-3.5 and ChatGPT-4, except for envy (*z* = −2.45, *p =* 0.01), where ChatGPT-4 achieved 100% correctness, whereas ChatGPT-3.5 was unable to give any correct answers. Similar to ChatGPT-3.5, ChatGPT-4 showed poor capabilities in responding to stories involving negative emotions, i.e., sadness and anger.

### 3.3 Comparison between ChatGPT-3.5 against human performance

**Figure 1:**
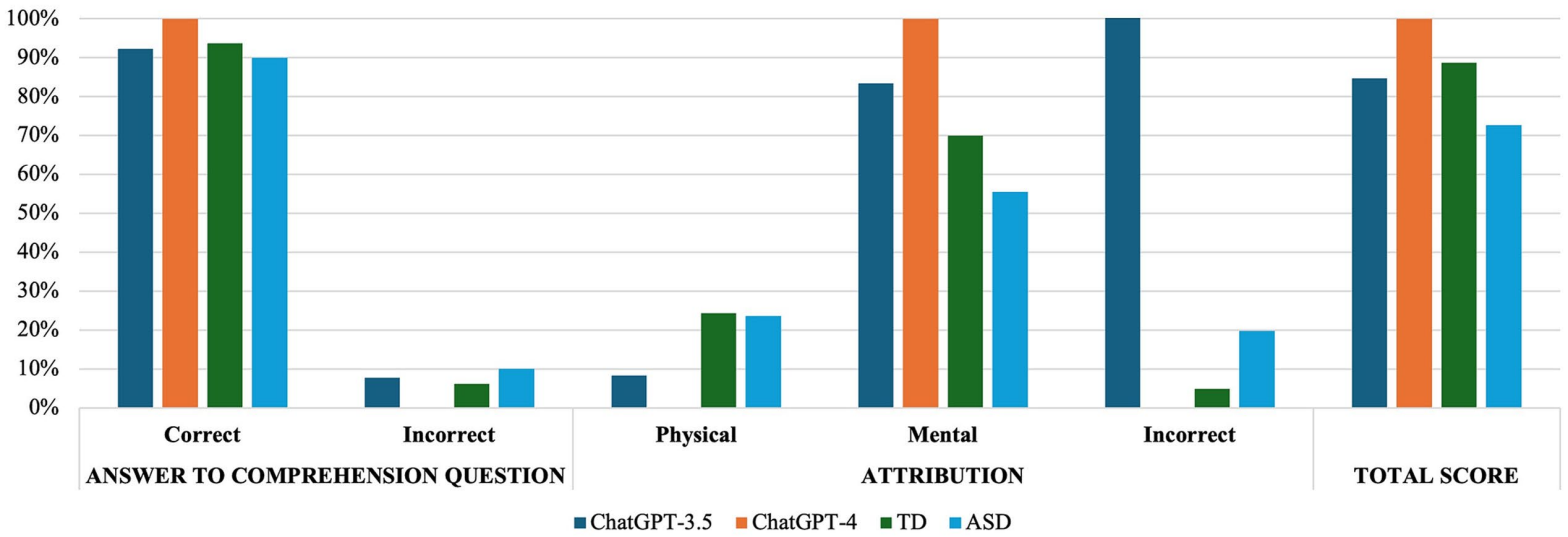
Response style used by ChatGPT and human groups in A-ToM.

The results of the binomial test showed no significant differences when comparing the accuracy proportions between the ChatGPT-3.5 and the TD group (*p* = 0.65) or the ASD group (*p* = 0.53) for the A-ToM. Figure 1 reports in detail the response style used by ChatGPT-3.5 and human groups.

**Figure 2:**
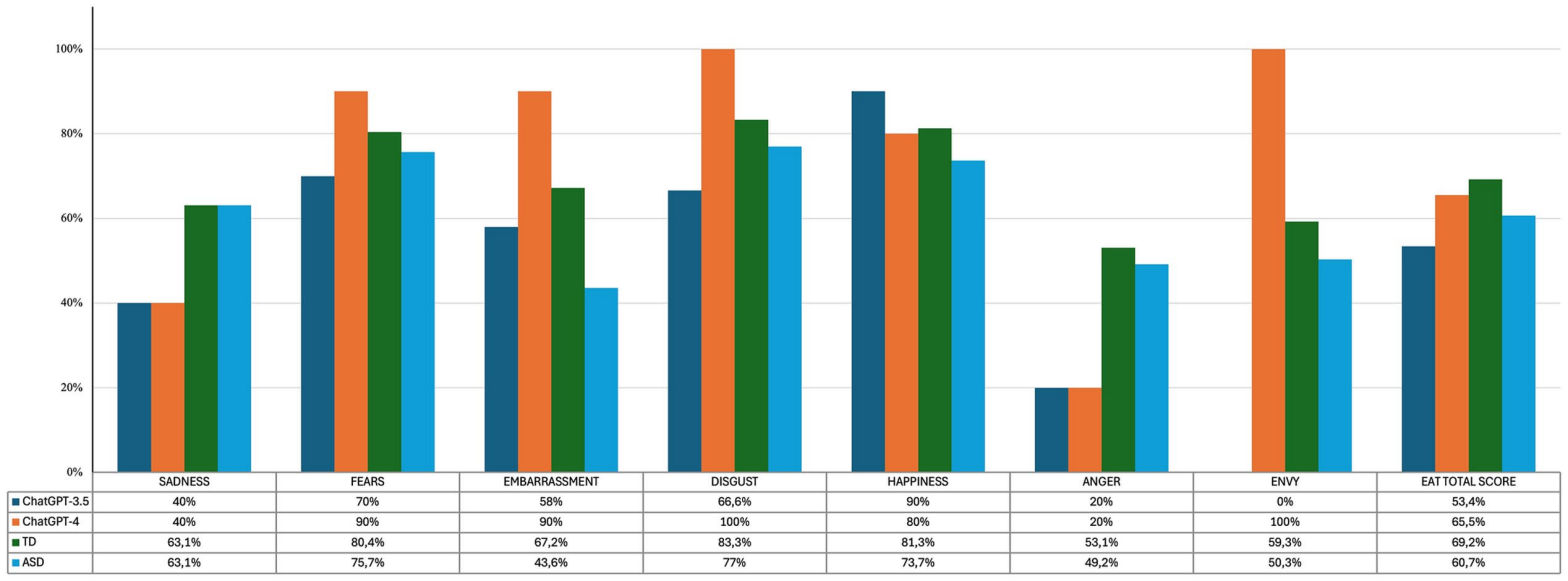
Percentages of correctness in the EAT test of ChatGPT-3.5, ChatGPT-4, TD and ASD.

Regarding the EAT test, ChatGPT-3.5 showed significantly lower accuracy than the TD group (*p* = 0.01) but did not differ from the ASD group (*p* = 0.28) in the Total Score. Furthermore, there was a trend toward significance in the anger accuracy rate of the ChatGPT-3.5 compared to the TD group (*p* = 0.053), but not compared to the ASD group (*p* = 0.11). No other differences were found between the ChatGPT-3.5 and the human groups. See Figure 2 for details.

### 3.4 Comparison between ChatGPT-4 against human performance

ChatGPT-4 accuracy proportions in the A-ToM did not differ from those of TDs (*p* = 0.39), while appeared significantly higher than those of ASDs (*p* = 0.02). Figure 1 reports in detail the response style used by ChatGPT-4 and human groups.

We found no difference between the accuracy proportions of TDs (*p* = 0.57) and ASDs (*p* = 0.50) compared with ChatGPT-4 in the Total Score of EAT. Also for ChatGPT-4, the anger scores showed a tendency to significance when compared with TDs (*p* = 0.053) but not with ASDs (*p* = 0.11). Finally, ChatGPT-4 accuracy proportion in embarrassment was significantly better than those obtained from the ASD group (*p* = 0.04). No other differences were found between ChatGPT-4 and the human groups. See Figure 2 for details.

## 4 Discussion

In recent years, the interaction between AI and humans has grown considerably, becoming part of multiple aspects of everyday life (Haque and Li, 2024). While the surprising abilities of LLMs to simulate human language open up critical reflections by raising ethical and moral questions (Aru et al., 2023), their potential application in various experimental fields cannot be ignored (Sartori and Orrù, 2023; Thirunavukarasu et al., 2023a). In this regard, some studies explored the strengths and limitations of LLMs as ChatGPT in educational, clinical, and mental health contexts (Dave et al., 2023; Haque and Li, 2024; Nori et al., 2023; Thirunavukarasu et al., 2023a, 2023b). Assuming that technological innovations in the field of mental health, when based on empirical evidence, constitute an added value, we wondered how LLMs simulate one of the fundamental human abilities for social and interpersonal interaction, namely ToM, which is known to be compromised in clinical populations such as ASD. For these reasons, we performed an affective and cognitive ToM test on two widely used LLMs, namely ChatGPT-3.5 and ChatGPT-4, by comparing their performance with those of TD and Level-1 ASD individuals. Our preliminary results provide important insights, including the hypothesis of applying this type of AI as support for individuals with high-functioning ASD. The first level of analysis was the comparison between TD and ASD individuals, and the results confirmed what has been reported in the literature (Baron-Cohen et al., 2001; Mazza et al., 2022, 2024; Polónyiová et al., 2024) with ASDs that performed worse than TDs on tasks that require inferring mental and emotional states from a social scenario. The second level of analysis included the performance of the LLMs and overall we found no statistically significant differences between ChatGPT-3.5 and ChatGPT-4 on the tests administered, although ChatGPT-4 achieved higher levels of accuracy than ChatGPT-3.5. In the A-ToM, both ChatGPT preferred the use of mental rather than physical attributions, however, unlike ChatGPT-4 which made no errors, ChatGPT- 3.5 responded correctly to all stories except two that investigated more complex mental states. In particular, ChatGPT- 3.5 missed the comprehension question in Story 2 which investigates “Persuasion” where a woman tries to convince a boy to take a kitten by telling him that if no one takes a cat, she will be forced to drown it. ChatGPT-3.5 responded as follows (translated from Italian): “*Ms. Rossi’s statement appears to be an attempt to persuade Giuseppe to adopt one of the kittens, using an emotional and dramatic tactic. We cannot determine the truth of this statement without more details, but the phrase “if no one buys the kittens I will be forced to drown them” seems to be a way of emphasizing a sense of urgency and necessity. Ms. Rossi may be overwhelmed by the amount of kittens she has to manage and may be worried about their future well-being if she cannot find homes for them. However, it is important to note that threatening to drown animals is unacceptable from an ethical standpoint and raises concerns about the treatment of animals. If Giuseppe is interested in getting a kitten, he should consider more humane and responsible options, such as adopting at an animal shelter or looking for organizations that can help find new homes for kittens. In addition, the situation highlights the importance of addressing feline overpopulation through spay/neuter and responsible adoption.”* ChatGPT-3.5’s response appears artificial and verbose, not seeming to capture the main core of the story, going “off-topic.” In addition, it shows ambiguity and remains anchored to the text, failing to conclude that the woman was not telling the truth, despite it recognizing the attempt at persuasion. Persuasion is a particularly complex state of mind in that it requires understanding that others have a different mental state that can be modified (Lonigro et al., 2017; Mazza et al., 2022). Moreover, persuasion involves manipulation, strategic deception, and guilt that seem to be too complex demands unless multiple social perspectives are adopted (Mazza et al., 2022). It’s interesting to note that persuasion is one of the stories in which individuals with ASDs seem to have more difficulties than TDs (Mazza et al., 2022). This trend also emerged in our sample, where 47% of the ASD participants failed the response. According to Mazza et al. (2022), the wrong answers of the participants in the ASD group could be linked to an inability to integrate all the information to achieve the overall sense and this explanation could also fit the performance of ChatGPT-3.5. The metallization style used by ChatGPT-3.5 also failed in the only story that investigates third-order ToM (Story 11-Double Bluff). In the story of the Double Bluff, Simon - a notorious liar - tries to deceive Max by pointing to the wrong place where he has hidden his racket. To answer correctly, it is necessary to understand the deception, go beyond the literal meaning, and make an inference about the mental state of the protagonist that, knowing that Simon is a liar, he will not go looking for the racket in the place indicated because he knows that Simon is certainly lying. ChatGPT-3.5 replied as follows (translated from Italian): *“No, it’s not true what Simon said to Max. Simon is known as a great liar, and so his answer could be a lie too. Max, knowing the behavior of Simon, may be skeptical about his brother’s response. However, Max might still decide to check under the bed because it is the specific location that Simon mentioned. Even though Max knows that Simon may be lying, Simon’s response could still influence his decisions about looking for the racket.”* In this case, ChatGPT-3.5 correctly answers to comprehension question, recognizes that Simon is a liar but does not correctly infer Max’s mental state, and consequently makes a mistake in indicating the final place where Max should look for the racket, which is the closet only. In our study, the Double Bluff represented the story that, after Persuasion, recorded the highest number of failures (39%) in the ASD sample. It is known that third-order ToM includes a rather complex level of recursivity, implying a meta-representation of the type “I think that you think that she/he thinks that another person thinks” (Happé, 1994; Mazza et al., 2022; Valle et al., 2015). To pass this type of task, it is first necessary to attribute a mental state to the character and, based on this, predict his/her behavior (Mazza et al., 2022; Valle et al., 2015). This ability is closely linked to social experience and requires, more than anything else, to go beyond the literal meaning, an aspect in which ChatGPT-3.5, similar to individuals with ASD, seems to fail. Unlike ChatGPT-3.5, ChatGPT-4 did not commit this type of error for the A-ToM and, as reported in the literature, achieves perfect levels overall (van Duijn et al., 2023). In our study, the performance of ChatGPT-4 in the A-ToM did not differ significantly from that of the TD group, whereas it appeared significantly better than that of ASD individuals. As suggested by Kosinski (2023), we cannot exclude the possibility that LLMs, especially those more advanced as ChatGPT-4, were repeatedly exposed to false belief tasks and, for this reason, learned the correct solutions during training.

One aspect that ChatGPT-3.5 and ChatGPT-4 have in common is the tendency to provide repetitive, verbose, and mechanical responses. The style adopted by ChatGPT, although in most cases leading to a correct answer for passing the test itself, appears long-winded, not always relevant to the topic, ambiguous, and not necessarily based on evidence provided by the context, thus violating Gricean conversational maxims (Barattieri di San Pietro et al., 2023; Grice, 1975; Marchetti et al., 2023). This evidence suggests that in mentalistic reasoning ChatGPT seems to differ significantly from the communications and mentalistic style used by TD individuals who in most cases give answers that are clear and concise (e.g., *No it’s not true, she says it to persuade Giuseppe to take one of the kittens*), reporting a sufficient and non-redundant amount of information (Maxim of quantity), based on mentalistic or physical inferences from the context (maxim of quality and relation), through clear and immediately comprehensible language (maxim of manner). In some ways, ChatGPT’s responses resemble the style used by individuals with high-functioning ASD who tend to provide irrelevant details or considerations (e.g., *Some people tell lies to try to convince other people*), ambiguous interpretations in mentalistic reasoning (e.g., *Ms. Rossi probably tries to create an anxiety-inducing customer experience as a last resource to get rid of the cats*), using explanations that involve reinterpreting the context to match the literal meaning (e.g., *because she cannot keep kitten*) or remain overly anchored to it (e.g., *Coming to a correct answer is difficult based on the evidence available to us*), often risking violating Grice’s maxims (De Marchena and Eigsti, 2016; Di Michele et al., 2007; Surian et al., 1996).

ChatGPT’s difficulty in adopting a clear and concise communicative style when faced with metallization tasks also emerges in the EAT test. To attribute emotions, both versions of ChatGPT needed further specification during the administration of the task and the explicit (additional) request to try to give a more precise answer about the emotion felt by the protagonist of the story. Despite the additional prompts, in many scenarios, ChatGPT provided at least three terms that referred to different emotions (fear, sadness, anger), or physical and mental states (frustration, fatigue, sense of betrayal, shock, defeat) rather than a single basic emotion. Similar responses also emerge in ASD individuals, including the tendency to confuse emotions with physical and mental states or to overlap emotions with negative valence. In this regard, our analyses showed that ChatGPT-3.5 performed worse than TD participants in identifying basic emotions but did not differ from ASDs. Most of ChatGPT-3.5’s errors were in identifying negative emotions such as sadness, envy, and especially anger, while on the contrary, it appeared more accurate in identifying happiness. Although further investigations are certainly needed, it could be hypothesized that this tendency is linked to the predilection of LLMs to generate responses with positive feelings, as they are pre-trained in this sense (Bian et al., 2023). According to Bian et al. (2023), this phenomenon reflects the human positivity bias known as the “Pollyanna Principle” (Boucher and Osgood, 1969). ChatGPT-4’s performance seems to be qualitatively better than ChatGPT-3.5’s although a specific difficulty remains in sadness and anger. These results are particularly significant as they suggest that understanding and attributing an emotional state may be a more complex task for an LLM than inferring a mental state based on contextual information alone (Banimelhem and Amayreh, 2023). It is well known that recognizing and understanding emotions plays an adaptive and survival function (Ekman and Davidson, 1994) and involves a deeper level of sharing with others that goes beyond verbal language, encompassing aspects such as facial expressions, body language, and previous socio-cultural experiences, which an AI lacks.

## 5 Conclusion

Our preliminary results show that in terms of accuracy, the performance of ChatGPT is halfway between that of TD and ASD individuals. The best results are recorded in cognitive ToM, although ChatGPT-3.5 fails in stories requiring the inference of more complex mental states. The ability to simulate human skills, including understanding emotions and using pragmatic language and an appropriate communicative style similar to that of a typical human being, remains one of the greatest challenges for LLMs. Further studies are needed that include different versions of ToM tests through repeated administrations over time and with different LLMs compared. We believe that AI cannot in any way replace the human being, especially in understanding mental and emotional states and social interaction. It can probably learn from the “verbal meaning of context” but it is still a simulation. Since ChatGPT’s performance has characteristics, on the one hand, similar to those of TDs and on the other hand very similar to those of a high-functioning ASD individual, this tool could represent a kind of “bridge” and be used for the advantage of people with ASD. Future research should investigate the possible applications of ChatGPT as a potential support and facilitation tool for people with autism, e.g., for decoding everyday and social life situations.

Our study has several limitations. The first is the sample size and gender distribution. While the primary aim of our study was not to compare the performance of human participants, the higher proportion of female participants in the TD groups limits the generalizability of our findings. Conversely, there is a male predominance in the ASD group compared to the TD groups, a common issue in ASD research, as the disorder involves approximately four males for every female (Valenti et al., 2019). Future studies should replicate these findings with a more balanced sample. A second limitation concerns the qualitative approach used to analyze the content responses of the human groups and LLMs (e.g., about Grice’s Maxims). Future research should investigate the communication strategies and pragmatic skills of LLMs more systematically. Studies on the cognitive and social skills of LLMs are still limited in the literature, and it would be useful to develop standardized methods to assess the cognitive abilities of chatbots. For instance, conducting repeated administrations of ToM tasks with ChatGPT could provide a sufficient number of observations for comparison with a sample of human subjects. Additionally, it would be useful to investigate the impact of different prompts, comparing in detail the variability of the ChatGPT’s responses. Although the preliminary nature of our study limits the generalizability of our results, it could provide valuable insights that warrant further investigation into the ToM abilities of LLMs in comparison to humans in future research.

## Data Availability

The raw data supporting the conclusions of this article will be made available by the authors, without undue reservation.
